# Can Sub-Zero Treatment at −75 °C Bring Any Benefits to Tools Manufacturing?

**DOI:** 10.3390/ma12233827

**Published:** 2019-11-21

**Authors:** Martin Kusý, Lýdia Rízeková-Trnková, Jozef Krajčovič, Ivo Dlouhý, Peter Jurči

**Affiliations:** 1 Institute of Materials Science, Faculty of Materials Science and Technology in Trnava, Paulínská 16, 917 24 Trnava, Slovakia; martin.kusy@stuba.sk (M.K.); jozef_krajcovic@stuba.sk (J.K.); 2 Institute of Physics of Materials, CEITEC-IPM, Czech Academy of Sciences, Zizkova 22, 61662 Brno, Czech Republic; idlouhy@ipm.cz

**Keywords:** vanadis 6 steel, sub-zero treatment at −75 °C, microstructure, hardness, fracture toughness

## Abstract

Vanadis 6 ledeburitic tool steel was subjected to sub-zero treatment at −75 °C for different durations, and for different subsequent tempering regimes. The impact of these treatments on the microstructure, hardness variations, and toughness characteristics of the steel was investigated. The obtained results infer that the retained austenite amount was reduced to one fourth by sub-zero treatment (SZT), and the population density of add-on carbides was increased by factor of three to seven, depending on the duration of SZT. Tempering always reduced the population density of these particles. A hardness increased by 30–60 HV10 was recorded after sub-zero treatment but tempering to the secondary hardness peak induced much more significant hardness decrease than what was established in conventionally quenched steel. The flexural strength was not negatively influenced by sub-zero treatment at −75 °C while the fracture toughness tests gave worse values of this quantity, except the case of steel tempered to the secondary hardness peak.

## 1. Introduction

In conventional heat treatment of tools made of high-alloyed Cr and Cr–V ledeburitic steels, the material is gradually heated up to the austenitizing temperature (recommended by the steel manufacturers), held there for prescribed time, and cooled down rapidly to the room temperature. Afterwards the steels should be subjected to tempering immediately, to prevent the retained austenite stabilization, and to induce transformations leading to the achievement of final properties of tools or components.

Ledeburitic steels are commonly used as tool materials for cold work applications. In order to generate high abrasive wear resistance, they contain a high amount of carbide phases embedded in a metallic matrix. On the other hand, these carbides together with high overall steel hardness deteriorate the material resistance against crack initiation/propagation, expressed by the fracture toughness K_IC_. Also, the flexural strength of ledeburitic steels, which is often also taken as a measure of resistance against crack initiation for brittle materials, manifests relatively low values. Moreover, conventionally produced ledeburitic steels are cast and afterwards hot formed. As a consequence, they contain band-like carbides, thus they suffer from anisotropy of key mechanical properties [1].

Sub-zero treatment is defined as a supplementary process to the conventional heat treatment. Unlike conventional heat treatment (CHT), it is a process where the tools or components are immersed into suitable cryoprocessing medium, stored there for pre-determined time (usually in tens of hours), and re-heated to the room temperature. Research works conducted on application of this kind of treatment have shown that sub-zero treatments provide extra benefits to the tooling industry like increased hardness [2,3], better wear performance [3,4], and improved dimensional stability of products [5].

According to recent studies, the following crucial microstructural changes are responsible for these benefits:

(i) Considerably reduced retained austenite (γ_R_) amount [2,6,7,8,9].
(ii) Refinement of the martensite along with an enhanced number of crystal defects such as dislocations and twins inside martensitic domains [7,9,10].
(iii) Enhancement of number and population density of small globular carbides (SGCs) [2,3,7,8,11,12].
(iv) Acceleration of the precipitation rate of nano-sized transient carbides [11,12,13,14,15].

However, the impact of sub-zero treatments on the toughness characteristics (fracture toughness, flexural strength, and impact toughness) is controversial as Table 1 illustrates.

The question of an optimal regime of sub-zero treatments is still under debate. In the "pioneer age" of this technique it was believed, within the professional community, that the benefits of sub-zero treatments are based only on the reduction of retained austenite amount. Therefore, the temperatures of around −75 °C were widely used in laboratory and industrial practice. Lower temperatures were not accepted for the treatments since their use often led to premature failure of tools, due to thermal shocks associated with the use of very low temperatures. Treatment at the boiling temperature of liquid nitrogen (−196 °C) was introduced into industrial practice only much later, when the devices enabled to carry out well controlled cooling down to such low temperature.

Other sub-zero treatment temperatures were suggested only by very limited number researchers. Reitz and Pendray and Gavriljuk et al., for instance, suggested the temperature of −140 °C [24,25]. Recent studies dealing with thorough analysis of microstructure and toughness of the Vanadis 6 steel treated in this way gave very promising results [22,26]. Alternatively, there were attempts and/or suggestions with the use of the temperature of boiling helium (−269 °C) [13,27]. However, the treatment temperature of −75 °C also deserves attention since one can expect that the phenomena being responsible for abovementioned ameliorations in properties would proceed faster at −75 °C than at lower temperatures. Also, practical experiences indicated that the extent of "extra" wear performance (or other property), which can be gained by the use of SZT at −196 °C (as compared with treatments at −75 °C) depends on the material chemistry. For instance, the wear performance of AISI D2 steel was improved by a factor of 2.59 by the treatment in liquid nitrogen (as compared with treatment at −75 °C), while only an improvement by a factor of 1.39 was recorded for CPM 10-V steel (steel with high vanadium content) [24].

The current paper is thus focused to an in-depth description of the results obtained with the sub-zero treatments of Vanadis 6 steel at the temperature of −75 °C, and to their careful discussion. Microstructural changes are presented, and they are related to the hardness, flexural strength, and fracture toughness of examined steel. The obtained results are also compared to what was obtained by treatments at temperatures −140, −196, and −269 °C, respectively.

## 2. Experimental

The powder metallurgy (PM) tool steel, Vanadis 6, with nominal composition (in wt%) 2.1 C, 1 Si, 0.4 Mn, 6.8 Cr, 1.5 Mo, 5.4 V, and Fe as a balance was selected for the examinations. Due to the PM technique used for the steel manufacturing the material is free of macrosegregations and also of carbide bands, and manifests high degree of isotropy. This makes it possible to disregard the orientation in sample manufacturing. The initial microstructure was soft-annealed, with a hardness of 272 HV10.

The specimens were machined to a net-shape and subjected to the heat treatment schedules. Conventional heat treatment consisted of gradual heating up to the austenitizing temperature of 1050 °C, holding at that temperature for 30 minutes to enable the dissolution of carbides and austenite homogenization, which was followed by room temperature quenching by using cold nitrogen gas. Then, one set of specimens was separated and subjected instantly to tempering treatments. The other specimens were moved to cryogenic system and subjected to sub-zero treatments at the temperature of −75 °C, and for different durations prior to tempering. 

In sub-zero treatments, room temperature quenched specimens were cooled down to −75 °C, at a controlled cooling rate of 1 °C/min, stored at the lowest temperature for predetermined duration (4, 10, 17, 24, or 48 h), and re-heated by a heating rate of 1 °C/min to the room temperature. Immediately after the specimens reached the room temperature, they were moved to the tempering furnace where they were tempered in an atmosphere of pure technical nitrogen. Tempering consisted of two cycles (2 + 2 h), at temperatures in the range 170–530 °C. However, the tempering temperature of 600 °C was added to the experiments with flexural strength, in order to verify the behavior of this mechanical quantity at tempering temperature that is located beyond the secondary hardness peak.

A NETZSCH DIL 402 C dilatometer (Netzsch, Selb, Germany) was used within the temperature range of 20 down to −150 °C in order to estimate the martensite finish temperature of the Vanadis 6 steel austenitized at given austenitizing conditions.

The specimens for microstructural examinations were prepared using standard metallographic grinding by a set of abrasive papers (with a grit of 180, 320, 600, and 1200) and polishing (by using the 9, 3, and 1 μm diamond suspensions). Then, the specimens were etched with Villella-Bain reagent (5 ml hydrochloric acid, 1 g picric acid in 100 ml of ethanol) for 10 s. For the material microstructure examinations, a JEOL JSM 7600 F scanning electron microscope (SEM, Jeol Ltd., Tokyo, Japan), operating at a 15 kV acceleration voltage was used. Microstructural examinations were coupled with energy dispersive spectrometry (EDS), by using an EDS detector (Oxford Instruments, plc., High Wycombe, UK). SEM micrographs were acquired in a combined 50:50 detection of secondary electrons and backscattered electrons (BE). The reason was that the material contains eutectic carbides (ECs), secondary carbides (SCs), and small globular carbides after application of abovementioned heat treatment schedules. It has been proven recently that the ECs are represented by vanadium-rich (more than 50 wt%V) MC particles, while the SCs are the M_7_C_3_ particles (with a high percentage of chromium), and that SGCs were determined to be alloyed cementite [7]. This made it possible to clearly differentiate between the ECs on the one side, and the SCs (and the SGCs) on the other side, by strong differences in BE yield. Unfortunately, this categorization fails in differentiation between SCs and SGCs as their BE yield is very similar. Therefore, the classification based on the particle size was adopted to distinguish the ECs and the SGCs; the carbides finer than 0.5 μm were considered as the SGCs while the coarser ones as SCs.

Determination of population density of carbide particles (categorized as above-described) was carried out on twenty-five randomly acquired SEM micrographs for each specimen. Standard magnification for acquisition of SEM images was 3000×. For better identification of SGCs additional SEM micrographs, at a magnification of 7500× were recorded because some of these particles had a size well below 100 nm. The acquisition of SEM images was coupled with EDS mapping of chromium and vanadium, in order to clearly differentiate between the carbides, as above mentioned. The mean values and the standard deviations of the obtained data were calculated.

The phase constitution of differently heat-treated specimens was determined by using X-ray diffraction (XRD, Philips Analytical B.V., Almelo, The Netherlands) technique. A Phillips PW 1710 diffractometer with filtered Co_Kα_ characteristic radiation was used for this purpose. The diffracted radiation was registered within a two-theta angle range of 30–127 deg. The retained austenite amounts were determined following the appropriate ASTM E975-13 standard [28], taking the characteristic peaks of both the martensite (α´) and the austenite (γ), namely (200)α´, (200)γ, (211)α´, and (220)γ into the consideration. As reported recently [2,7], however, these peaks are often superimposed by the characteristic peaks of carbides, which might influence the accuracy of the obtained results negatively. Therefore, the analyses were coupled with Rietveld refinement of the obtained X-ray spectra before computing the retained austenite amounts.

Hardness of differently heat-treated specimens was measured by using a Vickers indentation technique, at a load of 98.1 N (HV 10), following the appropriate Czech standard [29]. A ZWICK 3212 hardness tester (Zwick-Roell, Ulm, Germany) was used. The distance between two adjacent indents was kept minimum 5 mm, and the dwell time used for each indent was 15 s. Ten measurements were done for each specimen. Then, both the mean values and standard deviations of measured values were calculated.

10 by 10 by 100 mm bar specimens were used for flexural strength determination. Prior to measurements the specimens were polished to a final surface roughness, R_a_, between 0.05 and 0.07 μm. This step is very important to obtain reliable results because it is known that the surface finish plays an important role in this type of measurement, and differences in surface finish (in microns of R_a_) can influence the obtained flexural strength values within the range of hundreds MPa [30]. An Instron 8862 test device (Instron, Norwood, MA, USA) was used. Specimens were tested in three-point bending configuration, at a loading rate of 1 mm/min, until the moment of fracture. The distance between loading roller supports was 80 mm. The flexural strength R was calculated according to equation (following the appropriate Czech standard [31])

(1) $$R=\frac{3FL}{2bh^{2}}\;[\mathrm{MPa}]$$

where F represents fracture force (maximum load on the load–deflection trace), L is distance of roller supports in three-point bending, b is the specimen thickness, and h is the specimen height (dimension in the direction of the acting load). In addition, the total work of fracture, W_of_, and the plastic part of the work of fracture, W_pl_, were evaluated from the corresponding area below the measured load-deflection (load displacement) curve.

Reliable evaluation of fracture toughness of materials by plane strain fracture toughness requires prior fatigue pre-cracking of the specimens in order to achieve a sharp and reproducible crack tip geometry for testing. Therefore, pre-cracked specimens with 10 by 10 by 55 mm dimensions were used for fracture toughness determination in the current work. Pre-cracking the samples was carried out after the heat treatment. A resonance frequency machine Cracktronic 8024 (Russenberger Prüfmaschinen AG, Neuhausen am Rheinfall, Switzerland) was used for this purpose, and the specimens were loaded in four-point bending configuration. The crack development was monitored on both sides of the sample using a digital long-distance microscope. Both the pre-crack preparation and the testing were carried out at room temperature following the ISO 12137 standard [32]. In testing, the specimens were loaded in three-point bending with a roller span of 40 mm, and at a loading rate of 0.1 mm/min. An Instron 8862 machine (Instron, Norwood, MA, USA) was used. Specimen deflection was measured by means of an inductive transducer integrated directly into the loading axis. Five samples were tested for each investigated heat treatment (SZT, tempering) condition.

Fracture surfaces were analyzed by using the scanning electron microscope JEOL JSM 7600 F. SEM micrographs were acquired in the secondary electrons detection regime, at different magnifications enabling to study the micro-morphology of fracture surfaces. Particular attention has been paid to the role of carbide particles in the fracture propagation.

## 3. Results and Discussion

**Figure 1:**
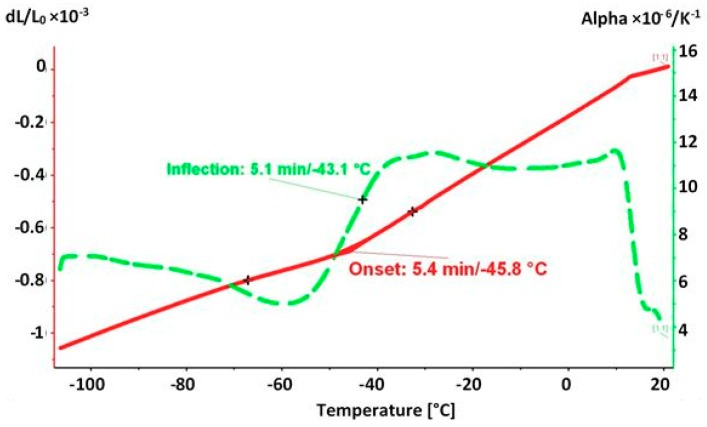
Dilatometry of Vanadis 6 steel after austenitizing at 1050 °C for 30 min, quenching to 20 °C, and cooling from 20 °C down to −110 °C with a cooling rate of 10 K min^−1^.

The change in the length of the Vanadis 6 specimen after quenching from 1050 °C down to 20 °C and immediate moving to the dilatometer, where the material was cooled down from 20 °C to −110 °C is presented in Figure 1.

The onset at the relative length curve is located at −45.8 °C. The inflection at the linear expansion coefficient curve is at −43.1 °C. From these two values it can be summarized that the critical M_f_ temperature of the Vanadis 6 steel lies at approximately −45 °C, it is thus higher than the selected sub-zero treatment temperature.

**Figure 2:**
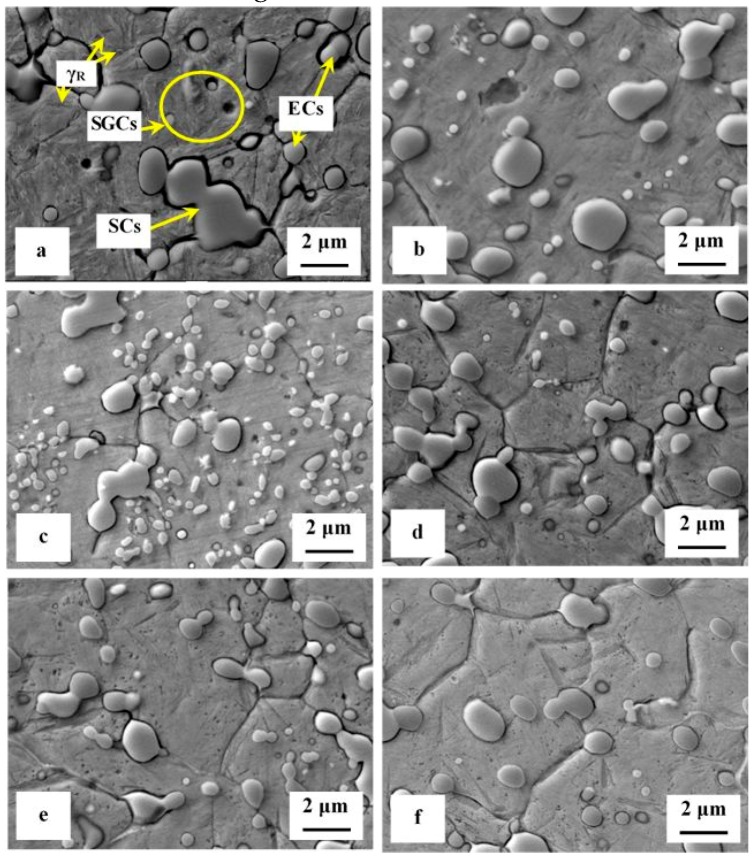
SEM micrographs showing the microstructure of no-tempered steel after conventional heat treatment (**a**), and after SZT at −75 °C for 4 h (**b**), 10 h (**c**), 17 h (**d**), 24 h (**e**), and for 48 h (**f**).

SEM micrographs, Figure 2, show the microstructure of Vanadis 6 steel in the conventionally heat-treated state (a), and after subsequent SZT at −75 °C for different durations (b–f). The matrix and different carbide particles are the main microstructural constituents of the steel, irrespectively to the heat treatment schedule used. The matrix is mainly martensitic, with certain amount of retained austenite. The retained austenite is located in-between the martensitic laths as SEM micrograph in Figure 2a illustrates. The carbides particles are the eutectic carbides, the secondary carbides, and small globular carbides. The number and population density of both the ECs and the SCs are nearly constant within the range of heat treatment schedules used. Alternatively, the number and population density of SGCs change with the duration of SZT; they increase substantially up to the 10 h duration of SZT, Figure 2b,c, where the maximum population density of 233 × 10^3^ mm^−2^ was achieved (Table 2), and then decrease, Figure 2d–f.

**Figure 3:**
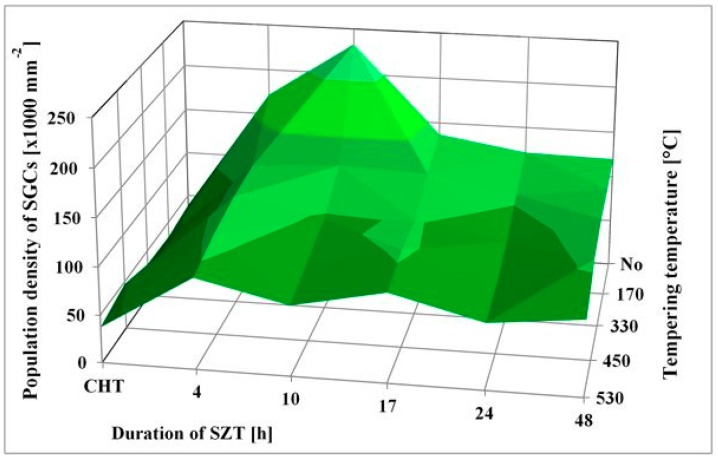
Population density of small globular carbides for differently sub-zero treated (at −75 °C) and tempered specimens.

It is shown in Table 2 and in Figure 3 that tempering treatment always induces a reduction of population density of SGCs. Despite that their population density remains two- to three-fold higher than what is produced by conventional heat treatment. Finally, it should be mentioned that SZT does not modify the amounts and population densities of ECs and SCs in Vanadis 6 steel as reported recently [7,12]. The reason is that these carbides are stable up to much higher temperatures and thus neither the SZTs nor the tempering do modify their quantitative characteristics.

**Figure 4:**
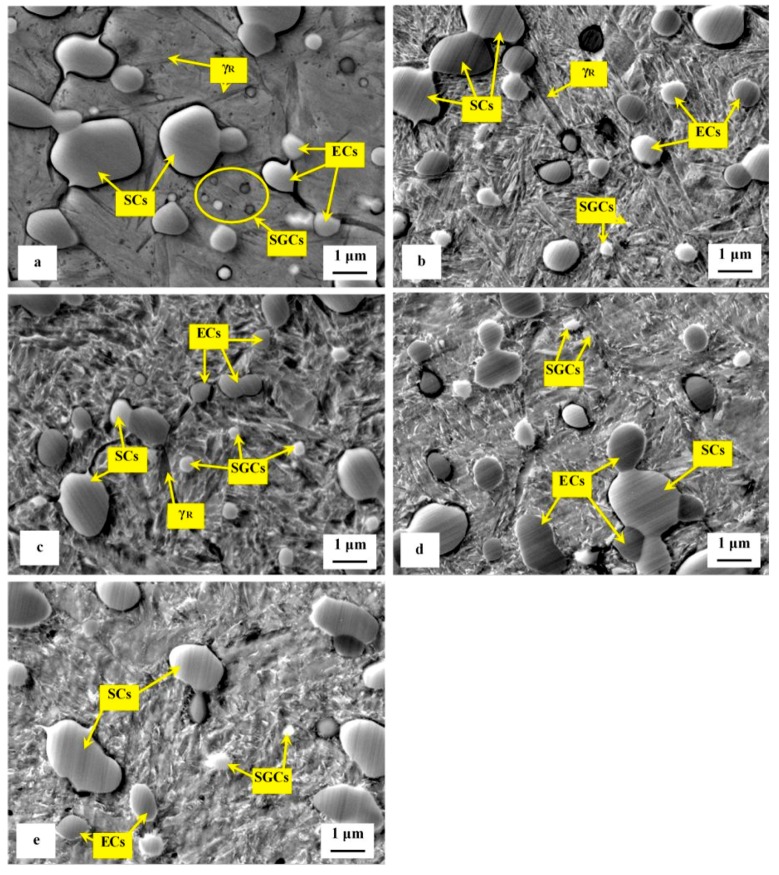
High-magnification SEM micrographs showing the microstructure of SZT steel (for 17 h) in the state after SZT (**a**), and after subsequent tempering at 170 °C (**b**), 330 °C (**c**), 450 °C (**d**), and 530 °C (**e**).

A series of high-magnification SEM micrographs, Figure 4, depicts the microstructural alterations of sub-zero treated (at −75 °C) steel with increasing the tempering temperature. The as-sub-zero treated material microstructure contains the matrix formed by the martensite and retained austenite and eutectic, secondary, and small globular carbides, Figure 4a. Tempering treatment modifies the material microstructure as follows: The matrix manifests more pronounced sensitivity to the etching agent, Figure 4b–e. This is reflected by extensive roughening of the originally smooth metallographic surface, and thus by lowered distinctness of originally clearly visible matrix microstructural features (martensite laths, retained austenite, grain boundaries etc.) obtained by austenitizing, quenching, and sub-zero treatments, compare with Figure 4a. Mentioned changes can be ascribed to extensive precipitation of nano-sized carbides. These carbide particles become visible after tempering at 450 or 530 °C, as tiny elongated formations, Figure 4d,e. At the same time the retained austenite disappears from the microstructure because it was decomposed into either "secondary" martensite or bainite during cooling down from the tempering temperature. Last but not the least, it should be noticed the population density of both the ECs and the SCs are nearly unaffected by tempering while the population density of SGCs decreases with tempering treatment, compare Figure 4a with micrographs in Figure 4b–e, and see also Table 2. It should be noted that similar microstructural development was detected also for the steel after SZT at −75 °C for other (shorter or longer) durations.

The dependence of the retained austenite amount on the tempering temperature for specimens after sub-zero treatment at −75 °C for 17 h is in Table 3. The amount of γ_R_ was 7.6 ± 0.4 vol% in the prior-to-tempering state. Tempering at low temperatures reduces the γ_R_ amount only moderately. In contrast, tempering at higher temperatures results in either the significant reduction (450 °C) or in the almost complete removal of the retained austenite. 

The γ_R_ amount in CHT specimens was around 20 vol% as reported recently [7,12]. The obtained results imply that the SZT at −75 °C reduces the γ_R_ amount to approximately one third as compared with the room temperature quenching. At the same time, however, it is worth to note that treatments at −140 or −196 °C act more effectively in reduction of retained austenite; the γ_R_ amounts were determined 4.3 and 2.1 vol% for steel that was SZT at −140 and −196 °C, respectively [7,12,26].

**Figure 5:**
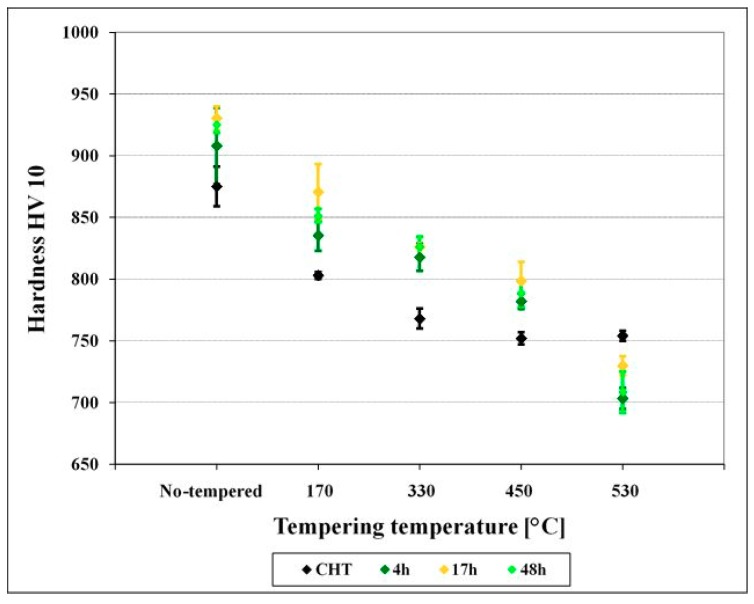
Hardness versus tempering temperature plots for conventionally heat-treated specimens and for specimens after sub-zero treatments at −75 °C for different durations.

The prior-to-tempering hardness for conventionally (room temperature) quenched Vanadis 6 steel was 875 ± 16 HV10, Figure 5. The hardness values of the steel after sub-zero treatment at −75 °C for 4, 17, and 48 h were 908 ± 30, 930 ± 10, and 925 ± 6 HV10, respectively. The obtained results, thus, imply that the hardness of the Vanadis 6 steel is higher due to the sub-zero treatments at −75 °C, and that the hardness improvement reaches the maximum for the material treated for the duration of 17 h. On the other hand, the hardness improvement is less significant than what was obtained by the treatment at lower SZT temperatures, e.g. at −140 or −196 °C, where values exceeding 950 HV10 were obtained [12,22].

The hardness of conventionally quenched steel was 803, 768, 752, and 754 HV 10 after tempering at temperatures of 170, 330, 450, and 530 °C, respectively. In other words, the hardness of conventionally heat-treated steel first decreases with increasing tempering temperature, and then it is preserved, almost constantly (at a level of 750 HV 10), when tempered at temperatures normally used for secondary hardening. In contrast, the steel after application of sub-zero treatments manifested higher hardness values, and this tendency was maintained up to the tempering temperature of 450 °C. The most significant hardness improvement was recorded for the steel treated for 17 h. For the specimens tempered at 530 °C, however, the hardness of sub-zero treated steel was lower than what was obtained by CHT.

These results are in line with those obtained by investigations of tempering response of the steel after SZT at −196 °C [12,20,21]. On the other hand, a strong variance between the tempering responses of the steel treated at −75 °C and the steel treated at −140 °C is evidenced. For the latter SZT temperature it has been reported recently that hardness of the material was improved significantly within the whole range of tempering regimes used [22].

**Figure 6:**
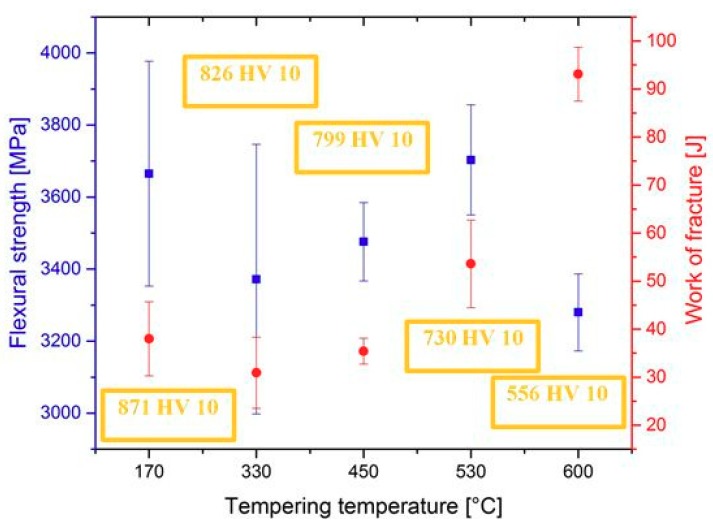
Flexural strength and work of fracture obtained by flexural three-point bend test for steel with application of SZT at −75 °C for 17 h, and subsequent tempering.

The flexural strength values obtained by three-point bend tests of samples having the microstructures according to Figure 4 are shown in Figure 6. It can be seen that the mean values of the flexural strength range between 3300 and 3700 MPa. The ranges of statistical uncertainty (at a probability level of 5%) overlap noticeably, suggesting that the tempering has only marginal effect on the flexural strength of SZT steel.

The work of fracture values of the steel tempered at 170, 330 and 450 °C lie within the range of statistical uncertainty, Figure 6, suggesting that low-temperature tempering does not influence this characteristic significantly. On the other hand, the work of fracture increases when the steel is tempered at temperature 530 °C and above. This can be associated with hardness decrease (as indicated in Figure 6), and thus with more extensive (compared to what occur during testing of specimens tempered at lower temperatures) plastic deformation of the matrix.

It is also well visible that the W_of_ values follow closely the values of flexural strength, but only up to the tempering temperature of 530 °C (peak of secondary hardness). For the specimens tempered at 600 °C, however, this relationship is no more valid. At this place it should be noted that the Vanadis 6 steel belongs to the group of brittle steels when tempered at temperatures up to the secondary hardness peak; in these cases, the obtained values of flexural strength are an indirect measure of its toughness. On the other hand, the flexural strength loses the characteristic of toughness when the hardness of the steel decreases. This is the case of "overtempering" of the steel, i.e., when the steel is tempered at temperatures beyond the secondary hardness peak, for instance at 600 °C. 

**Figure 7:**
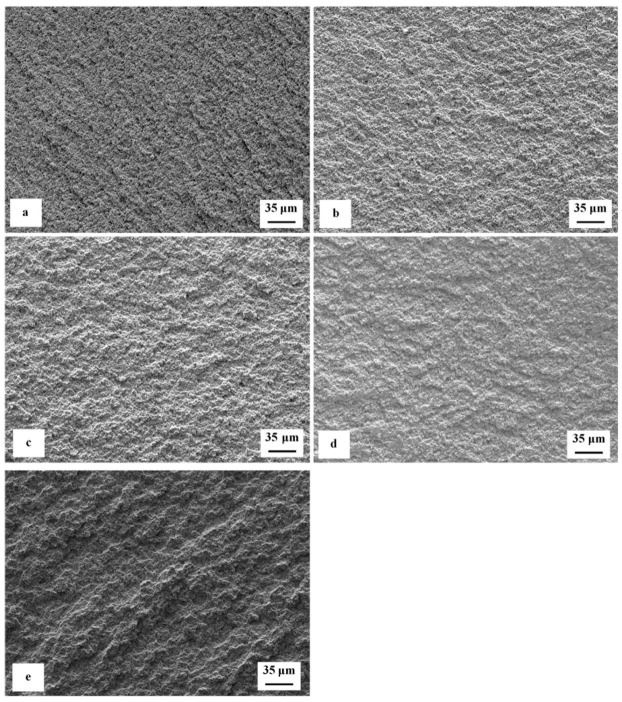
Representative SEM micrographs of the fracture surfaces of sub-zero treated specimens after flexural strength testing, (**a**) tempered at 170 °C, (**b**) tempered at 330 °C, (**c**) tempered at 450 °C, (**d**) tempered at 530 °C, and (**e**) tempered at 600 °C.

Figure 7 is a compilation of SEM micrographs showing fracture surfaces of flexural strength specimens that were sub-zero treated at −75 °C for 17 h and subsequently tempered at different temperatures. With respect to the surface morphology, the fracture surfaces can be divided into two groups. The fracture surfaces of the first group of specimens appear relatively flat, with only very limited roughness caused by strong difference between the fracture behavior of the matrix and carbides, Figure 7a–c. In contrast, the fracture surfaces (second group) of the specimens tempered at 530 and 600 °C (the latter one in particular) manifest much more pronounced indications of plastic deformation of the matrix, Figure 7d,e. Here it should be noted that the steel hardness decreased (and the plasticity expectedly increased) with increasing the tempering temperature, Figure 6. This correlates well with the above-mentioned morphology of fracture surfaces. In other words, the fracture surfaces appear relatively flat and shiny when the steel has higher hardness, but their topography increases with hardness decrease (i.e., with increasing the tempering temperature).

**Figure 8:**
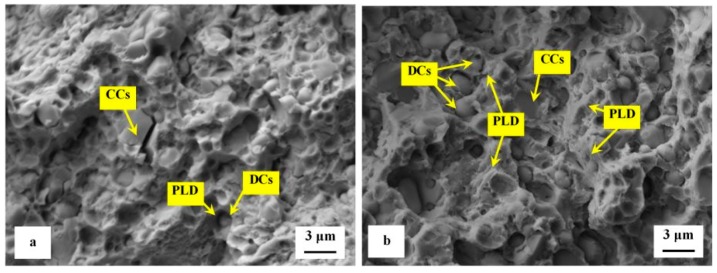
Detailed SEM micrographs of the fracture surfaces of sub-zero treated specimens after flexural strength testing, (**a**) tempered at 170 °C and (**b**) tempered at 600 °C.

Detailed SEM fractographs in Figure 8 clearly delineate the differences between the fracture surfaces obtained by testing of specimens tempered at 170 and 600 °C. A relatively flat morphology of the fracture surface with only very limited plastic deformation of the matrix is visible in Figure 8a. The presence of micro-plastic deformation is only visible at the sites where decohesion at the matrix/carbide interface took place during the crack propagation. The sites with local micro-plastic deformation are located mainly in close vicinity to smaller carbides, which act as decohesion sites at the above-mentioned interfaces. These carbides are denoted as decohesive carbides (DCs). Other carbide particles (the coarser ones, in most cases) are cleaved, and they are denoted as "cleaved carbides, CCs". In contrast to the fracture surface shown in Figure 8a the fracture of the specimen tempered at 600 °C manifests much more pronounced plastic deformation, Figure 8b. However, the role of particular carbides in the crack propagation is maintained and is almost the same as shown in Figure 8a.

**Figure 9:**
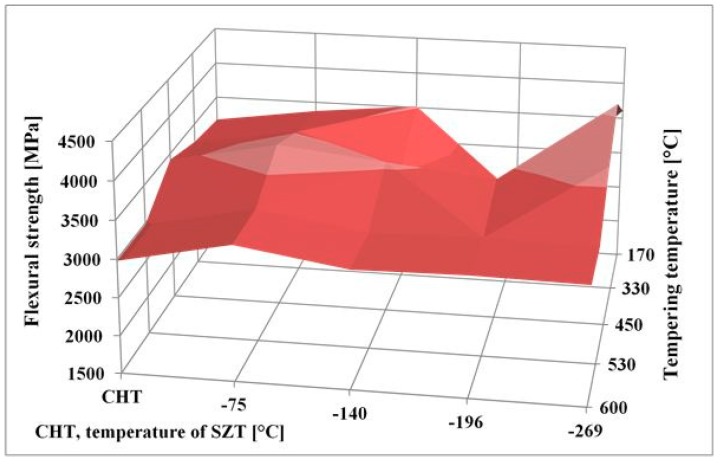
Comparison of the obtained flexural strength values for differently sub-zero treated and tempered specimens made of the Vanadis 6 steel.

The obtained flexural strength values for differently sub-zero treated and tempered specimens are summarized in Figure 9. It is shown that the treatment at −75 °C gave rather higher flexural strength than the conventional heat treatment. This is somewhat surprising at first glance, since one would expect a decrease in flexural strength rather than slight increase due to the application of SZT.

To explain this, it should be noted that Vanadis 6 steel contains 20.2, 7.6 4.8, 2.1, and 6.3 vol % of retained austenite after CHT, SZT at −75 °C, SZT at −140 °C, SZT at −196 °C, and SZT at −269 °C, respectively (all the SZTs were carried out for 17 h duration) [12,26]. Retained austenite is considered a soft microstructural feature, and one can expect its beneficial effect on toughness (and flexural strength). This may, for instance, partly explain the better flexural strength of the conventionally heat-treated material, as well as the material SZT at either −140 or −269 °C, than was obtained by SZT at −196 °C. However, this does not bring an answer to the improvement of flexural strength by SZT at −75 °C, compared to CHT steel. As mentioned above, sub-zero treatment at −75 °C produces a much higher population density of small globular carbides than CHT, which in turn leads to the formation of an increased number of matrix/carbide interfaces. The population density of small globular carbides decreases with tempering, despite remaining much higher than what can be obtained by CHT. In the steel samples with a higher population density of carbides, the crack propagation is more probably associated with micro-plastic deformation of a matrix (as Figure 8 clearly demonstrates); hence, an enhanced carbide count is an important factor responsible for improvements of flexural strength.

Finally, few words should be written to the fact that the treatments at either −140 or −269 °C lead to nearly equal or slightly better flexural strength than what is obtained in the present study. It has been reported recently that the treatment at −140 °C for 17 h results in the retained austenite amount of around 4.3 vol% [22]. The latest measurements fixed the γ_R_ amount to 6.3 vol% in the Vanadis 6 steel treated in liquid helium. This is two times (for −140 °C SZT) or three times (for −269 °C) more than what was obtained by SZT at −196 °C, but only of approximately 60% or 85% in comparison with the values reported here for SZT at −75 °C. One can thus expect rather opposite tendency with respect to the changes of flexural strength; however, the treatment at −140 °C (for instance) produces the greatest population density of SGCs, which more than fully compensates the toughness loss resulting from the reduction of retained austenite.

**Figure 10:**
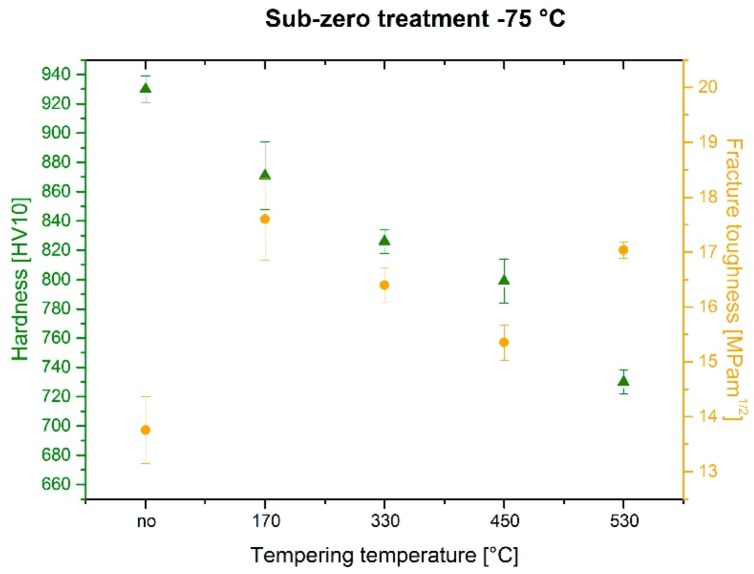
Fracture toughness versus hardness of the Vanadis 6 steel after SZT at −75 °C for 17 h, and subsequent tempering.

The relation between fracture toughness and hardness, as a function of tempering temperature, is in Figure 10. The of fracture toughness values were 13.76 ± 0.61, 17.60 ± 0.75, 16.40 ± 0.32, 15.35 ± 0.32, and 17.04 ± 0.15 MPa × m^1/2^ for specimens prior-to-tempering, tempered at 170, 330, 450, and 530 °C, respectively. The lowest K_IC_ values were determined for prior-to-tempering specimens, which had the highest hardness. This is logical because the hardness is the key factor that influences the fracture toughness, and it is generally accepted that metals with high hardness usually manifest low fracture toughness level, and vice versa [20,33]. The values of K_IC_ and hardness that were obtained by testing of tempered specimens do not obey this rule, however. It is for instance shown that the fracture toughness is high for the steel tempered at 170 °C despite its high hardness. Further, the K_IC_ decreases along with the hardness when the tempering temperature is increased, to either 330 or 450 °C. An increase in K_IC_ was recorded only for the steel tempered at 530 °C, but at lower a hardness value.

**Figure 11:**
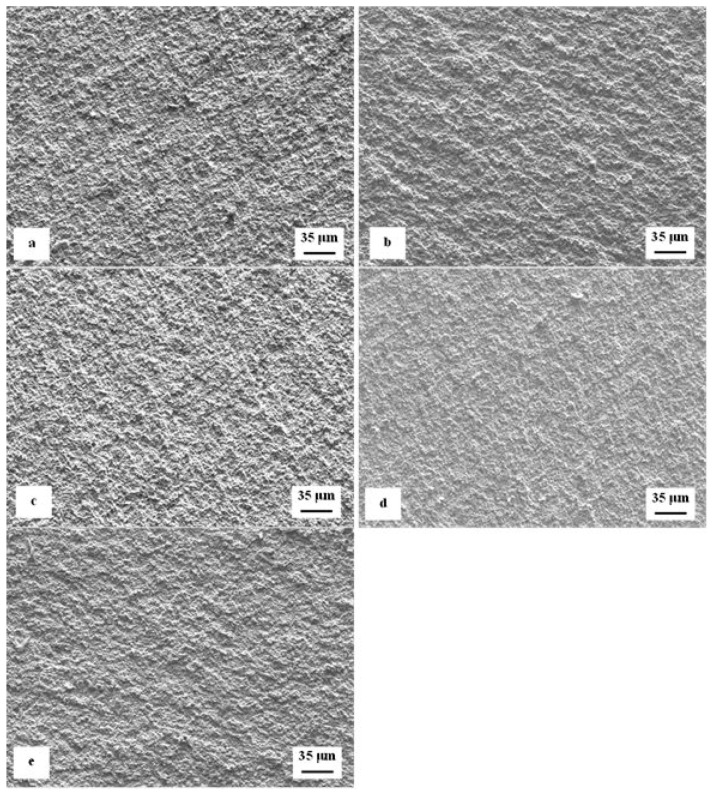
Representative SEM micrographs of the fracture surfaces of sub-zero treated specimens after fracture toughness testing, (**a**) no-tempered, (**b**) tempered at 170 °C, (**c**) tempered at 330 °C, (**d**) tempered at 450 °C, and (**e**) tempered at 530 °C.

The analysis of fracture surfaces provides a more comprehensive insight into the variations of fracture toughness with tempering. Figure 11 is a compilation of representative SEM micrographs of the fractured *K*_IC_ specimens that were SZT at −75 °C and no-tempered or tempered at different temperatures. The fracture surfaces of all the specimens manifest symptoms that are typical for hard and brittle steels; they appear flat, shiny, and relatively smooth. However, more thorough investigation reveals differences on the topography of fractured surfaces. The surface of no-tempered specimen (Figure 11a) manifests much finer topography compared with the fractured surfaces of other specimens (Figure 11b–e). This can be attributed to the differences in fracture toughness-no-tempered specimens have the lowest K_IC_ values, and tempering leads to moderate increase in this characteristic.

**Figure 12:**
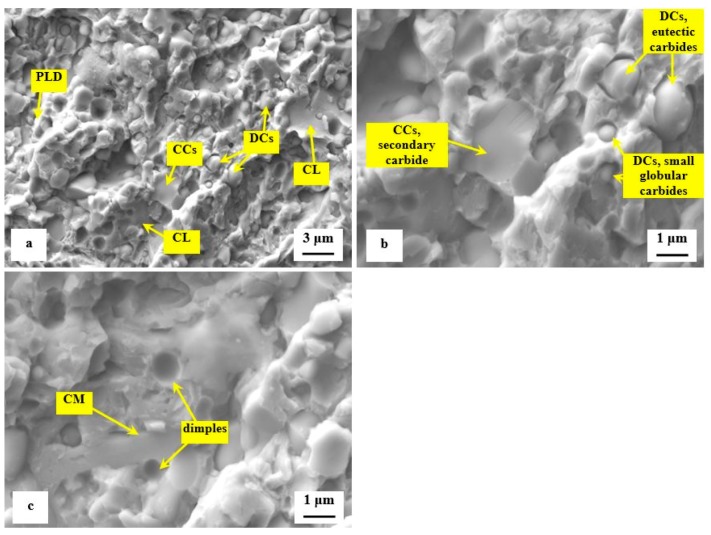
SEM images showing details of fracture propagation in sub-zero treated and no-tempered fracture toughness specimen, (**a**) detailed image showing the crack propagation in the matrix and the three main carbides including their role in fracture propagation, (CCs—cleaved carbides, DCs—decohesive carbides, PLD—plastic deformation in the matrix, CL—cleaved matrix), (**b**) high-magnification image showing cleaved M_7_C_3_ (CCs, secondary carbide), and decohesive particles that belong to eutectic carbides (DCs, eutectic carbides) and small globular carbides, and (**c**) high-magnification image showing cleaved matrix region (CM) and small dimples resulting from the extraction of small globular carbides.

Detailed SEM micrographs in Figure 12 were acquired from the specimen that was not tempered after sub-zero treatment. The image in Figure 12a assists in seeing that the fracture surface manifests typical morphology for hard steel. It contains a great number of micro-voids and holes, which correspond to extraction of SGCs (in particular) from the fracture surface during the crack propagation. The formation of micro-voids is associated with local plastic deformation of the matrix. However, the capability of the matrix to be deformed plastically is very limited, Figure 12b. There is a great number of sites with cleavage fracture mechanism apparent in the matrix as Figure 12c illustrates. The micrograph in Figure 12b also depicts the difference in behavior of carbides during the crack propagation. Some carbide particles (mainly the coarsest ones) were cleaved while others assisted the decohesion mechanism of the crack propagation. This is similar to what was discovered for the flexural strength specimens, Figure 8.

The role of particular carbides in the fracture propagation can be assessed with the help of recent investigations. It has been published recently that the coarsest particles belong mostly to the group of secondary carbides and that their nature is hexagonal M_7_C_3_ [7,11,12,22]. The finer particles are either eutectic carbides (MC-carbides with cubic crystallographic structure) or small globular carbides (cementite M_3_C) [7]. Casellas et al. clearly demonstrated that lower symmetry of the hexagonal crystalline lattice (as compared with cubic MC phase) results in low fracture toughness of M_7_C_3_, and correspondingly in large scatter of its values (0.5–4.5 MPa × m^1/2^) [34]. Moreover, Fukaura et al. [35] proved that the size of the carbides plays an important role in their fracture propagation manner, namely that coarser particles are much more amenable to the cleavage than the finer carbides. In Vanadis 6 steel, the mean spherical diameter of the M_7_C_3_ particles is of around 2.5–2.8 μm while the MC carbides have size within the range 1.6–1.9 μm [26]. The size of small globular carbides, SGCs, is even much smaller, below 0.5 μm. These facts assist to delineate the role of different carbides in fracture propagation as Figure 12 illustrates. Larger size and crystallographic anisotropy of M_7_C_3_ make this carbide more brittle than the MC (or M_3_C), despite that the hardness of M_7_C_3_ is lower than that of MC [34]. This is why the most part of cleaved carbides are the SCs (M_7_C_3_) while dominant number of ECs (and almost all the SGCs) is unaffected by the crack propagation, thus, assisting decohesion at the matrix/carbide interfaces.

**Figure 13:**
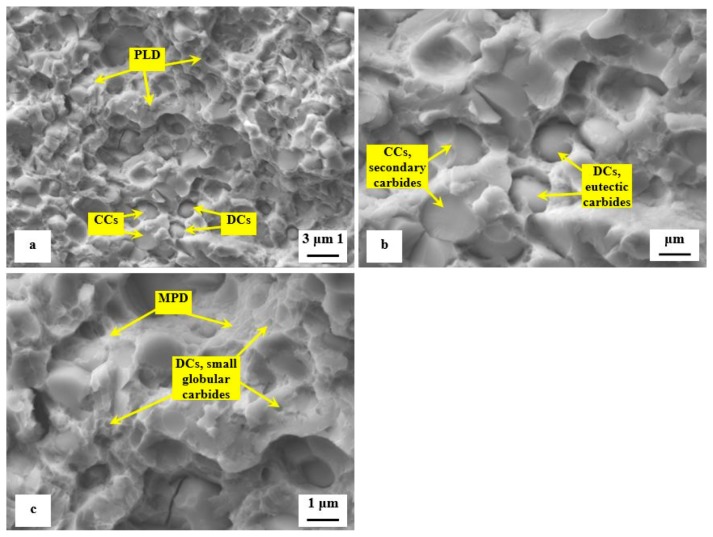
SEM images showing details of fracture propagation in sub-zero treated and subsequently tempered (at 530 °C) fracture toughness specimen, (**a**) detailed image showing the crack propagation in the matrix and the three main carbides including their role in fracture propagation, (CCs—cleaved carbides, DCs—decohesive carbides, PLD—plastic deformation in the matrix), (**b**) high-magnification image showing cleaved M_7_C_3_ (CCs, secondary carbide), and decohesive particles that belong to eutectic carbides (DCs, eutectic carbides), and (**c**) high-magnification image showing micro-plastic deformation of the matrix (MPD) and small dimples resulting from the extraction of small globular carbides.

The second series of detailed SEM micrographs, Figure 13, depicts the details of fracture propagation in the specimen that was sub-zero treated and subsequently tempered at 530 °C. It is worth noting that the fractured surfaces of specimens tempered at other temperatures did not differ significantly from that in Figure 13. Compared to the fracture surface of prior-to-tempering specimen, Figure 12, the fracture surface of the tempered one contains much greater area fraction of micro-plastically deformed matrix, Figure 13a. Further, there are cleaved carbides (CCs) and decohesive carbides present.

High-resolution SEM micrograph in Figure 13b shows details of cleaved secondary carbides (CCs) and decohesive eutectic carbide particles (ECs), suggesting that the role of particular carbides in the fracture propagation is very similar to the case of the prior-to tempering specimen, see Figure 12. In other words, the difference in fracture toughness (13.76 ± 0.61 MPa × m^1/2^ for the prior-to tempering steel versus 17.04 ± 0.15 MPa × m^1/2^ for the steel tempered at 530 °C) does not play a significant role in the fracture propagation mode of carbide particles. In contrast, mentioned difference in fracture toughness values is reflected in the matrix behavior. As Figure 13c illustrates, the fracture propagation on the matrix is associated with micro-plastic deformation, and the cleavage takes only a minor role.

**Figure 14:**
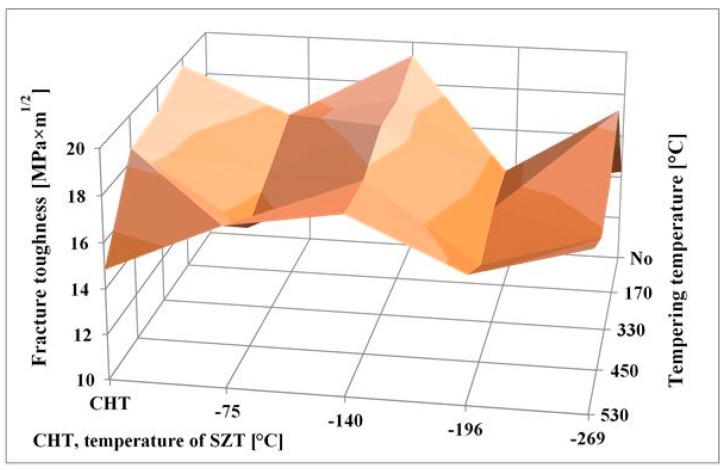
Comparison of the obtained fracture toughness values for differently sub-zero treated and tempered specimens made of the Vanadis 6 steel.

The differences in the fracture toughness, and correspondingly in the morphology of fracture surfaces of differently tempered specimens can be explained considering the material microstructure. Table 3 shows that the examined steel contains 7.6 ± 0.4 vol% of retained austenite in the prior-to-tempering state. The retained austenite is relatively soft, thus prone to plastic deformation. For instance, Putatunda [33] reported that the retained austenite has beneficial effect on the fracture toughness of high-carbon steels. However, the Vanadis 6 steel also contains high amount of hard and brittle "pre-aged" martensite in the prior-to-tempering state (it is worth noting that aging of the martensite in sub-zero treated high-carbon steels was many times experimentally proved, e.g. [14,15,26,36]), which makes the steel brittle. Berns and Broeckmann [1], Das et al. [19], and Ptačinová et al. [21] found out that the crack has a strong tendency to follow the interfaces between matrix and carbides when propagates during testing of ledeburitic steels. As a result, a micro-plastic deformation of the matrix occurs, which slightly improves the fracture toughness. Nevertheless, beneficial effects of retained austenite and increased population density of carbides cannot compensate the high brittleness of pre-aged martensite, hence, the material manifests very low fracture toughness as a consequence. Finally, it should be underlined that this finding is in line with the obtained results on the same steel, but processed at different SZT temperatures as Figure 14 illustrates. Finally, it should be underlined that this finding is in line with the obtained results by the K_IC_ testing of the same steel that was processed at other SZT temperatures as Figure 14 illustrates.

Tempering reduces the material hardness, Figure 5, and one can thus expect increase of fracture toughness. Increased fracture toughness was really recorded for sub-zero treated and subsequently tempered steel specimens, Figure 10. The clarification of this issue is relatively complex: On one hand the amount of soft retained austenite is either moderately reduced (for the steel tempered at 170 or 330 °C) or this phase is almost completely removed (after application of higher tempering temperatures), Table 3. Also, tempering reduces the population density of small globular carbides, Table 2, Figure 3. On the other hand, the martensite undergoes significant softening, which makes it more amenable to deform plastically. The resulting fracture toughness values are then a result of competition between mentioned three phenomena. The increase of K_IC_ values can be mainly ascribed to the martensite softening as this phase is the major one in the material. Increase in fracture toughness is reflected in more pronounced topography of fractured surfaces (and in more visible dimples on fracture surfaces at the same time), compare Figure 11a with Figure 11b–e, or Figure 12 with Figure 13. 

Very interesting comparison of the current results with those obtained by testing of CHT steel and the steel that was subjected to SZT at −140, −196, or −269 °C is provided in Figure 14. It is shown that the SZT at −140 °C gave the best results that are fully comparable with conventionally heat-treated steel, but at significantly increased hardness as reported in [22]. Treatments at either the temperatures of boiling nitrogen or helium resulted rather in lower fracture toughness values.

A reliable explanation of the variations in fracture toughness is very complex issue. In brief, the resulting fracture toughness value of ledeburitic tool steels is always a result of competition between three effects: i) retained austenite amount, which undoubtedly acts in favor of higher K_IC_, ii) state of the martensite (K_IC_ decrease), and iii) population density of small globular carbides (increase of K_IC_) [22]. CHT steel contains of around 20 vol% of the retained austenite in the prior-to-tempering state, and the retained austenite amount is nearly constant up to the tempering temperature of 500 °C [12]. Even though the population density of SGCs is very low in CHT steel (as compared with the steel after SZTs) it is more than satisfactorily compensated by the retained austenite amount. Application of SZTs reduces the retained austenite amount considerably (with the minimum value for SZT at −196 °C) and increases the population density of SGCs (with the maximum for the SZT at −140 °C). The results in Figure 14 imply that the reduction of retained austenite is almost fully compensated by much higher population density of SGCs for the steel after SZT at –140 °C while other SZTs do not lead to high enough population density of carbides needed to compensate the impact of reduced retained austenite amount on the resulting K_IC_ value.

Finally, it should be noted that these considerations do not take into account the state of the martensite. In the state after CHT (and prior-to-tempering) the martensite was found to be "pre-aged", but it did not contain any carbide precipitates [7]. In contrast, the martensite after SZTs contained nano-sized, coherent transient carbides ([12,26]. However, it is hard or practically impossible to provide an exact assessment of the difference between impacts of these two martensite states on the fracture toughness of the steel since it also contains the retained austenite and several carbide types. The situation is very similar in the case of tempered steel Vanadis 6 because tempering leads to acceleration of precipitation rate at low-tempering temperatures but rather to delayed precipitation at high temperatures (around 500 °C) [11,12,26]. But, an exact quantification of precipitates is almost impossible.

## 4. Conclusions

The impact of sub-zero treatment at the temperature of −75 °C and subsequent tempering on the microstructure, hardness, flexural strength and fracture toughness was investigated. The main obtained results can be summarized as follows:

- The population density of small globular carbides is increased by application of SZT at −75 °C, by the factor from the range 2.5 and seven.
- Retained austenite amount is reduced by this kind of treatment, to an approximately one third as compared with conventional room temperature quenching.
- Third bullet
- The bulk hardness of the steel is increased by SZT at −75 °C, by 30–60 HV10. Improved steel hardness is maintained up to the tempering temperature of 450 °C while tempering at 530 °C leads to more significant hardness reduction than what is obtained by conventional heat treatment.
- The flexural strength of SZT steel ranges between 3300 and 3700 MPa. The level of tempering temperature has only little impact in the flexural strength.
- The fracture toughness of sub-zero treated no-tempered steel is very low. However, it increases with the tempering application, and reaches relatively high values of around 17 MPa × m1/2 after tempering to the secondary hardness peak.
- In summary, the application of SZT at −75 °C may bring some benefits into heat treatment of tool steels. However, the obtained microstructures and values of mechanical properties are lower as compared, for instance, with those obtained by treatment at −140 °C.

## Figures and Tables

**Table 1:** Toughness and fracture toughness of differently sub-zero treated ledeburitic steels—an overview of the obtained results to date.

Steel	Treatment	Quantity	Description	Reference
AISI D2	−70, −100, −130, and −196 °C, tempering at 200 °C	Impact toughness	Considerable reduction, extent of reduction depends on both the austenitizing temperature (better toughness is achieved at lower austenitizing temperature) and SZT temperature (−70 °C produces the lowest values)	[16]
AISI D2	−196 °C/16 h, tempering at 170 or 450 °C	Impact toughness	Considerable reduction, degree of reduction is much higher than the improvement of hardness	[17]
AISI D2	−196 °C/20 h	Impact toughness	Reduction after low-temperature tempering but improvement after tempering to the secondary hardness	[18]
AISI D2	−196 °C/36 h, −75 °C/5 min −125 °C/5 min, tempering at 210 °C	Fracture toughness	Significant reduction after SZT at either –75 or –125 °C, moderate reduction after SZT at –196 °C	[19]
Vanadis 6	−196 °C/4 or 10 h, −90 °C/4 h, tempering twice at 530 °C	Flexural strength, fracture toughness	Marginal effect on flexural strength but slightly positive effect on fracture toughness	[20]
Vanadis 6	−196 °C/17 h, tempering 170–530 °C	Flexural strength, fracture toughness	Marginal effect on flexural strength in low-temperature Tempering range, improvement in normal secondary hardening temperature range. Worsening of fracture toughness except tempering to the secondary hardness.	[21]
Vanadis 6	−140 °C/17 h, tempering at 170–530 °C	Flexural strength, fracture toughness	Slight improvement of flexural strength at low tempering temperatures but almost no effect after tempering at 450 °C and above. Improvement of fracture toughness after high temperature tempering but slight deterioration after tempering within the 170–450 °C range	[22]
AISI D3	−196 °C/12, 24 or 36 h, tempering at 150 °C	Impact toughness	Significant reduction, the reduction is more pronounced with increasing the duration of SZT	[23]

**Table 2:** Determined values of population density of small globular carbides for differently sub-zero treated and tempered specimens.

Duration of SZT [h]	Tempering Temperature [°C]				
	No	170	330	450	530
CHT	38.7	33.5	35.3	47.6	37.4
4	171.6	119.4	116.1	107.9	95.4
10	233.2	125.6	97.0	84.5	73.5
17	136.9	121.9	101.7	103.0	93.3
24	121.8	104.6	84.8	73.7	69.2
48	119.8	115.6	115.4	106.0	79.5

**Table 3:** Retained austenite amount (in vol%) in Vanadis 6 steel after quenching followed by sub-zero treatment at −75 °C for 17 h, no-tempered and tempered at different temperatures.

Tempering Temperature [°C]	No-Tempered	170	330	450	530
Retained austenite amount [vol%]	7.6 ± 0.4	5.5 ± 0.1	4.6 ± 0.2	2.5 ± 0.1	Not measurable
